# Diagnostic Stability of Psychiatric Disorders in Re-Admitted Psychiatric Patients in Kerman, Iran

**DOI:** 10.5539/gjhs.v6n5p294

**Published:** 2014-07-15

**Authors:** Fatemeh Alavi, Nouzar Nakhaee, Abdolreza Sabahi

**Affiliations:** 1 Psychiatric Department, Kerman Medical University, Kerman, Iran; 2 Neuroscience Research Center, Kerman Medical University, Kerman, Iran; 3 Religion & Medicine Research Center, Kerman Medical University, Kerman, Iran

**Keywords:** diagnostic stability, psychiatric disorder, Iran

## Abstract

**Background:**

Several studies have evaluated the stability of psychiatric diagnosis follow in readmission of patients in psychiatric hospitals. However, there is little data concerning this matter from Iran. This study is designed to evaluate this diagnostic stability of the commonest psychiatric disorders in Iran.

**Objectives:**

The objective of this study was to determine the long-term diagnostic stability of the most prevalent psychiatric disorders among re-admitted patients at the Shahid Beheshti teaching hospital in Kerman, Iran.

**Patients and Methods:**

This study was based on 485 adult patients re-admitted at the Shahid Beheshti hospital between July and November 2012. All of the diagnoses were made according to DSM IV TR. Prospective and retrospective consistency and the ratio of patients who were obtained a diagnosis in at least 75%, 100% of the admissions were calculated.

**Results:**

The most frequent diagnoses at the first admission were bipolar disorder (48.5%) and Major depressive disorder (18.8%). The most stable diagnosis was bipolar disorder (71% prospective consistency, 69.4% retrospective consistency). Schizoaffective disorder had the greatest diagnostic instability (28.5% prospective consistency, 16.6% retrospective consistency).

**Conclusions:**

Among the cases evaluated, bipolar disorder had the most stability in diagnosis and the stability of schizoaffective disorder was poor.

## 1. Introduction

Mental disorders are among the leading causes of disease burden and the relevant disabilities worldwide. (Baca-Garcia et al., 2007).

Diagnosis provides some information about symptoms, treatment and prognosis of disease in patients, and also illustrate border for research among homogenous group of patients (Chang, Chan, & Chung, 2009).

In general, medicine worldwide diagnoses are based on International Classification of Disease (ICD), but in psychiatric diagnoses moreover the ICD, being used criteria of Diagnostic & Statistical Manual (DSM) for psychiatric disorder press of American Psychiatric Association.

Validity & reliability of diagnostic criteria debates on many researches based on diagnostic stability of psychiatric disorders.

The definition of psychiatric diagnosis mainly drives from expert opinion not biological bases (Baca-Garcia et al., 2007).

Unlike other branches of medical science that diagnoses are based on identification of the underlying biological procedure, still in psychiatry, the diagnosis is based on clinical syndromes identification (Chang et al., 2009).

There are no specific biological markers for evaluating the validity of diagnoses in psychiatry, then the longitudinal follow up is a method for evaluating the validity of them (Hollis, 2000).

The stability of diagnosis refers to consistency or difference between preliminary and follow up examination of patient and is used to determine response to treatment and to examine progression of disease (Atwoli et al., 2012).

The stability of diagnosis is the grade in which a diagnosis remains unchanged in consecutive examination of the patients; it is an index for evaluating the reliability of patient assessment (Whitty et al., 2005).

Diagnostic instability is very important because it has an impact on treatment planning and psychosocial interventions (Whitty et al., 2005).

Clinically, incorrect diagnosis can lead to inappropriate treatments and iatrogenic effects on the patients (Chang et al., 2009).

The belief is that if upcoming edition of DSM and ICD significantly improve the criteria of its predecessor, then the validity of diagnostic concepts will progress (Baca-Garcia et al., 2007).

Several studies have evaluated the stability of diagnosis in re-admitted patients in psychiatry (Jakobsen, Hansen, & Werge, 2007; Bulow & Svensson, 2004; Veen et al., 2004; Jabrin, & von Knorring, 2003; Amin et al., 1999), whilst there is very little study from Iran.

In a two years follow up study in Korean patients unstability was reported in three major psychiatric diagnoses; major depressive disorder, bipolar I disorder (BID) & schizophrenia. Of course upper stability was BID (Kim, Woo, Chae, & Bahk, 2011).

In study from south India modestly high stability in Acute & transient psychotic disorder (based on ICD criteria) was reported & few percent of these patient go to schizophrenia & bipolar disorder diagnosis (Janardhanan, Narayana, Virupaksha, Dhanaya, Biju, & Kesavan, 2012).

In Bromet study shift of diagnosis to schizophrenia (32%) and then to bipolar disorder (10.7%) has revealed that is to indicate of misclassification in first admission (Bromet et al., 2011).

A research in Iran (Tehran) reported high stability of schizophrenia & bipolar disorder diagnosis (Amini et al., 2005).

The aim of our study was to evaluate the long-term stability of the most prevalent chronic psychiatric diagnoses according to DSM IV TR, in Kerman; southeast of Iran.

## 2. Method

This study was conducted between July and December 2012 at the Shahid Beheshti hospital in Kerman, Iran. This study was a historical cohort study over a period of six months. It was based on 485 adult patients (age 18 years and over) with at least one previous psychiatric admission & 2530 episode admission in summation. All diagnoses were made on the basis of DSM IV TR criteria, for the period of 12years (2000-2012). Data was then collected from the patients´ previous medical records, including socio demographic data, date of previous admission, diagnoses in previous admission and given treatment (e.g. ECT). The collected data was analyzed using the statistical package for social science (SPSS ver. 20).

Four criterias were evaluated for each diagnosis. The first criteria was prospective consistency that is the proportion of patients in a diagnostic category at the first evaluation who received the same diagnosis at their last evaluation. That is equivalent to positive predictive value.

The second criteria was retrospective consistency that is the proportion of patients with a given diagnosis at the last admission who had received that at the first admission, this is equivalent to sensitivity.

The third criteria was the ratio of patients who received a particular diagnosis in at least 75% of the admissions. (Baca-Garcia et al, 2007) The forth criteria was the proportion of patients who received the same diagnosis in 100% of the evaluations.

## 3. Results

In total 485 cases were enrolled in the study, mean ±age of participants was 38.6±12, 7 and 71.1% of them were male. Socio demographic characteristics of the sample are presented in Table 1.

**Table 1:** Socio demographic characteristics

**Gender**	
Male	345(71.1)
Female	140(28.9)

**Marital status**
Single	186(38.4)
Married	250(51.5)
Divorced	38(7.8)
Widowed	11(2.3)

**Education**
Illiterate	77(15.9)
Under high school diploma	276(56.9)
High school diploma	99(20.4)
University	33(6.8)

In this sample, 51.5% were married, 38.4% were single, 7.8 were divorced and 2.3% were widowed.

The majority of participants had under high school diploma (56.9%).

The mean number of admission was 3.5 (2-21, s.d.5.3)

The mean days of each admission was 21.4

The most frequent diagnosis at the first admission was bipolar disorder (45.1%)

The majority of the patients on total admission were diagnosed, with bipolar disorder (48.5%), too (Table 2).

**Table 2:** Diagnoses at first admission and at total admission

Diagnoses(n=485)	Frequency at first admission (%)	Frequency at total admission (%)
**Bipolar disorder**	219(45/1)	1224(48/5)
**Schizophrenia**	59(12/1)	330(13/0)
**Schizoaffective disorder**	21(4/3)	173(6/8)
**Major depressive disorder**	92(18/9)	352(13/9)
**Anxiety disorder**	5(1/0)	43(1/7)
**Delusional disorder**	4(0/8)	14(0/5)
**Personality disorder**	45(9/2)	221(8/7)
**Substance related disorders**	40(8/2)	153(6/0)

The prospective consistency at the first admission was 71% for bipolar disorder, 55.9% for schizophrenia and 41.6% for major depressive disorder.

The retrospective consistency at the last admission was 69.4% for bipolar disorder and 68.6% for major depressive disorder. Schizoaffective disorder had the lowest prospective and retrospective consistency (See Table 3).

**Table 3:** Prospective and retrospective consistency of diagnoses

Diagnoses(n=485)	Prospective consistency (%)	Retrospective consistency (%)
**Bipolar disorder**	71.0	69.4
**Schizophrenia**	55.9	45.8
**Schizoaffective disorder**	28.5	16.6
**Major depressive disorder**	41.6	68.6
**Anxiety disorder**	50.0	55.0
**Delusional disorder**	50.0	40.0
**Personality disorder**	61.1	67.3
**Substance related disorders**	68.2	59.5

The ratio of patients who received a particular diagnosis during at least 75% of their admissions ranged from 74.7% for bipolar disorder to 9.5% for schizoaffective disorder (See Table 4).

**Table 4:** The proportion of patients who received a particular diagnosis at least 75% and 100% of admissions

Diagnosis	Patients who received a diagnosis in at least 75% of the admissions		Patients who received a diagnosis in 100% of the admissions	

	Number	%	Number	%
**Bipolar disorder**	148	74.7	114	57.6
**Schizophrenia**	34	41.4	21	36.2
**Schizoaffective disorder**	2	9.5	1	4.8
**Major depressive disorder**	35	47.9	23	31.5
**Anxiety disorder**	3	60	1	20
**Delusional disorder**	1	25	1	25
**Substance related disorders**	18	46.2	16	23
**Personality disorder**	13	36.1	5	31

The ratio of patients who received a particular diagnosis at 100% of their admissions was 57.6% for bipolar disorder and 4.8% for schizoaffective disorder.

## 4. Discussion

Diagnostic changes over time can indicate the evolution of a disease, as well as the presence of new information or unreliable diagnostic measurements (Schwartz et al., 2000).

Spitzer et al. (1987) categorized the causes of unreliability, leading to diagnostic disagreement among physicians to such categories (source of variance): subject variance (changing in the patients), occasions variance (different episodes of an illness), information variance(different informants or more information available during the follow up evaluation), observation variance (different interpretations of the same sign and symptoms) and criterion variance (two observers use two groups of criteria for diagnosis of an illness) (Baca-Garcia et al., 2007; Jabrin & von Knorring, 2003).

Information and observation variances can be improved through proper training of clinicians in interview and related techniques (Baca-Garcia et al., 2007).

Considering Table 2, bipolar disorder showed higher frequency than other diagnoses. This issue represents that bipolar disorder can be over diagnosed. Several studies have suggested the same subject (Zimmerman, Ruggero, Chelminski, & Young, 2010). On the other hand in our study, bipolar disorder showed relatively higher prospective and retrospective consistency than other studies. Dramatic representation of manic episodes can facilitate its diagnosis compared to other psychiatric disorders (Atwoli et al., 2012).

A significant point is that many psychiatrists make the first diagnosis of schizophrenia most conservatively. As such a diagnosis imposes much burden to the patients and their family. In our study the prospective and retrospective consistency of schizophrenia was lower than other studies, mostly done in industrial societies. There is a tendency to under diagnose schizophrenia in developing countries such as Iran.

An additional factor that should be mentioned is the presence of substance abuse or dependence. In many cases, it can change the clinical presentation of psychiatric disorders. In our study substance related disorders were the most prevalence co morbidities with other diagnoses such as schizophrenia, mood disorder, anxiety disorder and personality disorder.

The patients who received the diagnosis of substance related disorder, in first admission, should be followed carefully, because a major part of them shifts to another psychiatric disorder that unlike the first diagnosis, they need long term interventions and treatments (Whitty et al., 2005).

On the other side substance withdrawal treatment excludes insurance coverage in Iran, so some of these patients feign level of psychiatric disorders to use insurance coverage. The differential diagnosis among mood disorder and personality disorder especially borderline personality disorder is difficult and complex, as referenced in textbook of psychiatry. The patients with personality disorder prone to axis I psychiatric disorder and during episodes of such diagnoses, personality disorder not well examined. The levels of personality disorder, in some clusters, tended to reduce over time. The reduction of personality disorder representations over time can be seen among patients with substance abuse or dependence, indicating that substance related disorders can mimic personality disorder (Ferro, Klein, Schwartz, Kasch, & Leader, 1998).

Another important matter that should be mentioned is the frequency of ECT in patients. It should be noted that repeated ECT can impact illness manifestations and course of the disease. Instability in the diagnosis of schizoaffective disorder indicates that the diagnostic criteria for definition of this group of patients are inefficient. Schizoaffective disorder has polymorphic patterns and during the course of illness, there are syndromes that change between schizoaffective disorder, pure mood disorder and pure schizophrenia. Focusing on cross sectional symptoms and lack of attention to the patients’ previous history make diagnosis of schizoaffective disorder more complicated (Chang et al., 2009).

The main strength of this study was the relatively large sample. The other strength is long term historical follow up; 12 years observation as long as durability of text-revised 4th edition of DSM that is relatively few in other studies.

The stability of psychiatric diagnoses has been evaluated in several studies. Most of these studies have been limited on the diagnosis of psychoses, especially schizophrenia, but we evaluated the stability of the most common diagnoses in psychiatry.

The other strength of this study was that the data were gathered from previous records of the patients in routine examinations. The patients were examined in neutral conditions.

The main weakness of our study was the retrospective follow–up.

Another weakness was the access of psychiatrists to patients’ previous records during the evaluations. It is recommended that they receive the same diagnosis especially if the previous interventions and treatments were successful.

## 5. Conclusions

In this study, the most frequent diagnosis was bipolar disorder and the less frequent diagnosis was delusional disorder. Among the diagnoses evaluated bipolar disorder had the most stability based on prospective and retrospective consistency and the stability of schizoaffective disorder was poor. The proportion of patients who received the same diagnosis in all of the admissions was between 4.8 and 57.6 percent (in schizoaffective disorder and bipolar disorder, respectively).
